# Acceptability of a Microbiome-Directed Food for the Management of Children with Uncomplicated Acute Malnutrition in Maradi, Niger: Two Randomized Crossover Trials

**DOI:** 10.1016/j.cdnut.2025.107484

**Published:** 2025-06-09

**Authors:** Susan M Rattigan, Ibrahim Ngoumboute Mbouombouo, Mohamed Antar Abdou Tahirou, Ishita Mostafa, Kazi Nazmus Saqeeb, Souna Garba, Ousmane Guindo, Tahmeed Ahmed, Michael J Barratt, Jeffrey I Gordon, Christopher R Sudfeld, Rebecca F Grais, Sheila Isanaka

**Affiliations:** 1 Department of Nutrition, Harvard T.H. Chan School of Public Health, Boston, MA, United States; 2 Epicentre, Niamey, Niger; 3 Nutrition Research Division, International Centre for Diarrhoeal Disease Research, Dhaka, Bangladesh; 4 The Edison Family Center for Genome Sciences and Systems Biology, The Newman Center for Gut Microbiome and Nutrition Research, St. Louis, MO, United States; 5 Department of Pathology and Immunology, Washington University School of Medicine, St. Louis, MO, United States; 6 Department of Global Health and Population, Harvard T.H. Chan School of Public Health, Boston, MA, United States; 7 Epicentre, Paris, France

**Keywords:** acute malnutrition, microbiome-directed food, acceptability, Niger, community-based management of acute malnutrition

## Abstract

**Background:**

A novel ready-to-use microbiome-directed food (MDF) has been developed for the management of acute malnutrition using ingredients that promote repair of the gut microbiota of undernourished children.

**Objectives:**

This study aims to assess the acceptability of MDF compared with standard nutritional care among children with acute malnutrition.

**Methods:**

Two randomized crossover trials consisting of 2 14-d periods of at-home consumption were conducted. Children aged 6 to <24 mo with severe acute malnutrition (SAM) or moderate acute malnutrition (MAM) were individually randomized in a 1:1 ratio to the sequence of receiving MDF then standard nutritional care, or vice versa. Standard nutritional care consisted of ready-to-use therapeutic food for SAM and ready-to-use supplementary food for MAM. The primary outcome was at-home acceptability, defined as the return of ≥75% of sachets empty after the 14-d at-home consumption period. The primary analysis was a noninferiority analysis, in which MDF was considered noninferior if the lower bound of the 95% confidence interval (CI) of the difference in at-home acceptability comparing MDF with standard nutritional care was within −20 percentage points. Secondary outcomes included caregiver’s perception of the child’s liking, as well as caregiver willingness to use in the future and preference between the 2 foods.

**Results:**

In all, 128 children with SAM and 146 children with MAM were randomized. MDF was noninferior to standard nutritional care in terms of at-home acceptability among children with SAM (risk difference: −7.0; 95% CI lower bound: −11.6%) and among children with MAM (risk difference: −2.3%; 95% CI lower bound: −6.1%). There were no differences in caregiver willingness to use either food in future.

**Conclusions:**

MDF is acceptable for the management of acute malnutrition in children aged 6 to <24 mo in Niger and should be further tested in other populations with a high prevalence of acute malnutrition. Effectiveness of the novel food will be assessed in forthcoming trials.

**Trial registration number:**

This trial was registered at clinicaltrials.gov as NCT05551819.

## Introduction

Acute malnutrition, classified as moderate [defined as a weight-for-length *Z*-score (WLZ) <−2 and ≥−3 or a mid-upper arm circumference (MUAC) of <125 and ≥115 mm] or severe (defined as WLZ < −3, MUAC < 115 mm or bilateral pitting edema), affects ≥45 million children each year with important consequences for child health and long-term development [1,2]. Standard care for acute malnutrition involves medical management and nutritional treatment for children with severe acute malnutrition (SAM) and nutritional supplementation for children with moderate acute malnutrition (MAM) [3]. Since 2007, the community-based management of uncomplicated acute malnutrition has allowed for the provision of ready-to-use therapeutic or supplementary foods for home use, with weekly or biweekly facility-based visits for clinical and anthropometric surveillance [4]. Although high rates of recovery can be achieved in community-based management [[5], [6], [7]], a high risk of relapse and death following successful discharge has been observed in some settings [8,9].

The high risk of relapse and mortality following programmatic discharge suggests that adaptations of the current formulation of standard nutritional care may better support improved longer term growth and development. A potential point of intervention may be the gut microbiome, a key interface for extracting and metabolizing nutritional components of food that is related to the clinical presentation of acute malnutrition [10,11]. In a study characterizing the gut microbiome in healthy and malnourished children in Bangladesh, children with SAM were shown to have a more immature microbiome compared with healthy children of a younger age [12]. A subsequent analysis of the gut microbiota in Malawi further found that implantation of the microbiome of malnourished children into gnotobiotic mice led to weight loss, changed bone morphology, and metabolic abnormalities, compared with the microbiome of healthy children [10]. These early studies provide support for a causal link between an immature microbiome and the pathogenesis of acute malnutrition.

To explore the potential of the microbiome as a practical target for improved growth and development in malnourished children, a microbiome-directed food (MDF) has been developed by the International Centre for Diarrheal Disease Research, Bangladesh in collaboration with Washington University in Saint Louis [13]. The formulation was developed by first testing 14 diets composed of commonly consumed Bangladeshi complementary foods in gnotobiotic mice that had been colonized with gut bacterial strains from Bangladeshi children. Recipes were sequentially tested in gnotobiotic mice, gnotobiotic piglets, and finally in 63 Bangladeshi children aged 12–18 mo with MAM, with a final formulation of MDF found to improve levels of biomarkers associated with neurodevelopment, bone development, and immune function, in a 1-mo pilot study among the children with MAM [13]. A subsequent 3-mo proof-of-concept study comparing MDF with standard nutritional care [ready-to-use supplementary food (RUSF)] in 123 Bangladeshi children aged 12–18 mo with MAM further demonstrated that MDF promoted microbiome repair, increased the mean weekly growth rate of WLZ, MUAC, and weight-for-age *Z*-score, increased plasma levels of biomarkers of musculoskeletal growth and neurodevelopment, and improved length-for-age *Z*-score in the 2 y posttreatment follow-up period compared with RUSF [14,15].

To determine the acceptability of MDF compared with standard nutritional care, we conducted a 2 × 2 crossover acceptability trial comparing MDF with standard ready-to-use therapeutic food (RUTF) among children with SAM and with standard RUSF among children with MAM aged 6 to <24 mo in rural Niger. Results will be used to improve MDF acceptability prior to large-scale trials among children aged 6 to <24 mo that will assess the clinical effectiveness of MDF compared with standard nutritional care in the management of SAM and MAM.

## Methods

### Study setting

Niger, a landlocked country of the Sahel in West Africa, consistently ranks among the least developed countries in the world [16]. Emergency levels of acute malnutrition were documented in 12.2% of children aged <5 y across Niger in 2022 [17]. Since 2009, health centers in Niger have provided outpatient management of uncomplicated acute malnutrition under a national protocol for the community-based management of acute malnutrition [18]. As per the national protocol, children aged 6–59 mo are eligible for SAM management with WLZ < −3, MUAC < 115 mm, or the presence of mild or moderate bipedal edema. Children are eligible for MAM management if they do not meet criteria for enrollment in SAM management and have a WLZ < −2 or MUAC < 125 mm. Children are excluded from outpatient management if they have insufficient appetite or clinical complications requiring hospitalization. Follow-up occurs on a weekly basis for children with SAM and on a biweekly basis for children with MAM. At each follow-up visit, medical history is obtained, and a physical examination including an anthropometric assessment is performed. Children remain in outpatient management until they achieve nutritional recovery or for 12 wk, whichever occurs first. Nutritional recovery is defined as WLZ ≥ −2, MUAC ≥ 125 mm, and no edema for 14 d.

### Trial design

This study included 2 at-home feeding trials for children with SAM and children with MAM, each employing a 2 × 2 crossover trial design. The design of the trials was informed by the 2019 UNICEF technical expert meeting that was convened to provide guidance on quality standards, product specifications, and evidence requirements for a new generation of RUTF formulations to be used in nutrition programs [19]. In the present trials, children were given a novel MDF (PRAN Agro Ltd) and standard nutritional care (standard RUTF for children with SAM or standard RUSF for children with MAM; Nutriset) during 2 14-d periods of at-home consumption, with each 14-d period preceded by an observed test dose of the food to be consumed. Children were individually randomly assigned to 1 of 2 feeding sequences (MDF then standard nutritional care, or standard nutritional care then MDF) in a 1:1 ratio. Randomization was stratified by sex and age group (6 to <12 and 12 to <24 mo). An independent statistician performed randomization using a computer-generated random number list. Blinding was not feasible because of the distinct packaging of standard nutritional care and MDF.

Inclusion criteria for the trials included children who were *1*) newly admitted to outpatient management of SAM or MAM, *2*) aged 6 to <24 mo, *3*) resided in the study catchment area (Tibiri and Sae Saboua health centers in Madarounfa, Niger) and caregiver planned to remain for ≥1 mo and *4*) had no known allergy or contraindication to ingredients of the study food or standard nutritional care. Written informed consent was obtained from the child’s legal guardian prior to enrollment. Caregivers were informed that MDF was an investigational product for the treatment of acute malnutrition that targeted gut health and contained chickpea flour, soy flour, peanut paste, green banana, oil, sugar, and micronutrients.

### Study procedures

#### Observed test dose

Prior to each 14-d at-home consumption period, an observed test dose of 20 g of the randomized food was given at the study health center. The test dose was weighed and subsequently provided to the caregiver to feed to the child. Administration of the observed test dose ended when either *1*) the entire dose was consumed, *2*) 30 min passed, or *3*) the child refused the food 3 times. Child refusal was defined as the child turning away, crying, clamping their mouth shut, or refusing to swallow. The remaining food was weighed to determine the amount consumed during the test dose. Mothers were asked to provide rankings of their perception of the child’s liking of the food on a Likert scale from 1 (dislike a lot) to 5 (like a lot) [20].

#### At-home consumption

After each test dose, caregivers were provided enough sachets of their randomized food to last until the next follow-up visit. Dosages of MDF (100 g/sachet), RUTF (92 g/sachet), and RUSF (100 g/sachet) were weight-dependent to provide 65 kcal/kg/d for children with MAM and 170 kcal/kg/d for children with SAM, as per the national protocol [18]. Caregivers of children with SAM were advised to feed only the study food and no at-home foods, and caregivers of children with MAM were advised to feed the study food along with the current home diet. For all children, continued breastfeeding was encouraged throughout the study, and study staff recommended that breast milk be offered and consumed before any study food. As an indirect measure of acceptability, caregivers were asked to return to scheduled study visits with all sachets provided at the prior visit, which study nurses recorded as completely used, partially used, or not used. Caregivers were additionally asked to rate their perception of their child’s liking of the food during at-home use on a 5-point Likert scale. After the first 14 d of at-home consumption, the child changed to the second study food according to their randomized feeding order without a washout period.

### Study outcomes

The primary outcome was food acceptability during at-home consumption. As direct observation at home was not feasible, returned sachet count was used as a proxy measure of consumption, as recommended by UNICEF [19]. The number of sachets returned used by the caregiver was divided by the number of sachets provided to determine the proportion consumed. A fully empty sachet returned was considered 1 consumed sachet; a partially empty sachet returned was considered 0.5 consumed sachet; sachets returned full or not returned were considered not consumed. Acceptability was defined as return of 75% or more of the sachets provided empty (yes/no).

Secondary outcomes during the at-home feeding included caregiver report of child’s liking, caregiver’s willingness to use the food in the future, sharing of the study food, and times per day the food was consumed. Liking and willingness to use the food in the future were defined as caregiver's report of a score of ≥4 on a 5-point Likert scale. Caregivers were asked which food they preferred at the end of the 28-d follow-up and 1 open-ended qualitative question to further explain their stated preference. Secondary outcomes during the observed 30-min test dose included acceptability of the observed 30-min test dose (defined as consumption of 75% or more of the 20 g observed test dose as weighed), result of test dose (dose finished within 30 min, 30 min elapsed, or food refused 3 times), and caregiver report of child’s liking (Likert scale of 1−5).

### Sample size

We calculated the sample size required to detect noninferiority of the acceptability of MDF compared with standard nutritional care for acute malnutrition. We assumed 75% acceptability of standard nutritional care and a noninferiority margin of an absolute −20 percentage difference. We assumed 90% power at a significance level of *α* = 0.025 using a 1-sided noninferiority test of correlated proportions, given the crossover design and anticipated 10% loss to follow-up. Given these parameters, 146 children with SAM and 146 children with MAM were required for each noninferiority analysis. Sample sizes were calculated using PASS Sample Size v14.

Enrollment of children with SAM was ended early because of slower than expected enrollment after the local harvest season when risk of malnutrition declines. Ad hoc power calculations that applied the observed acceptability from children with SAM enrolled up to that time indicated that a power of 90% had been achieved at the time of stopping.

### Statistical analysis

Statistical analyses were conducted separately for the SAM and MAM trials. The primary analyses in both trials were noninferiority analyses of at-home acceptability. MDF was considered noninferior to standard nutritional care if the lower bound of the 1-sided confidence interval (CI) of the estimated risk difference (RD) was less than the noninferiority margin of a −20-percentage point difference. The RD and 1-sided CI were calculated using generalized estimating equations with an identity link and Poisson variance function [21]. All secondary analyses were superiority analyses. Generalized estimating equations (GEE) models with a log link and binomial variance function were used to calculate relative risks (RR) and 95% CIs for binary secondary outcomes. If the log-binomial models did not converge, log-Poisson models, which provide consistent but not fully efficient estimates of the RR and its CIs, were used [22]. GEE models with an identity link and Gaussian variance function were used to calculate mean differences and 95% CIs for continuous secondary outcomes. All primary and secondary models included covariates for period and sequence effects and a compound symmetry covariance structure to account for the crossover design [[23], [24], [25]]. Models also included covariates for sex and age group to account for stratified randomization [26]. If log-Poisson models did not converge, covariates for age and sex and the sequence effect were sequentially removed until the model converged. A complete case analysis was performed for each outcome: children who completed the test dose for each food were included in analysis of test dose outcomes, and children with SAM who completed ≥1 wk of at-home consumption of each food and children with MAM who completed 2 wk of at-home consumption with each food were included in analysis of at-home consumption outcomes. Caregivers whose children completed all 4 wk of follow-up were asked a single open-ended question about their preferred food at the end of the 28-d follow-up period. Statistical significance was defined at *P* < 0.05, and no adjustments were made for multiple comparisons. Analyses were run in R version 4.3.2.

### Ethics

The trial was conducted in accordance with Good Clinical Practice guidelines. The study protocol was approved by the Comité Consultatif National d’Ethique (Niamey, Niger) and the Harvard Longwood Campus Institutional Review Board.

## Results

**FIGURE 1:**
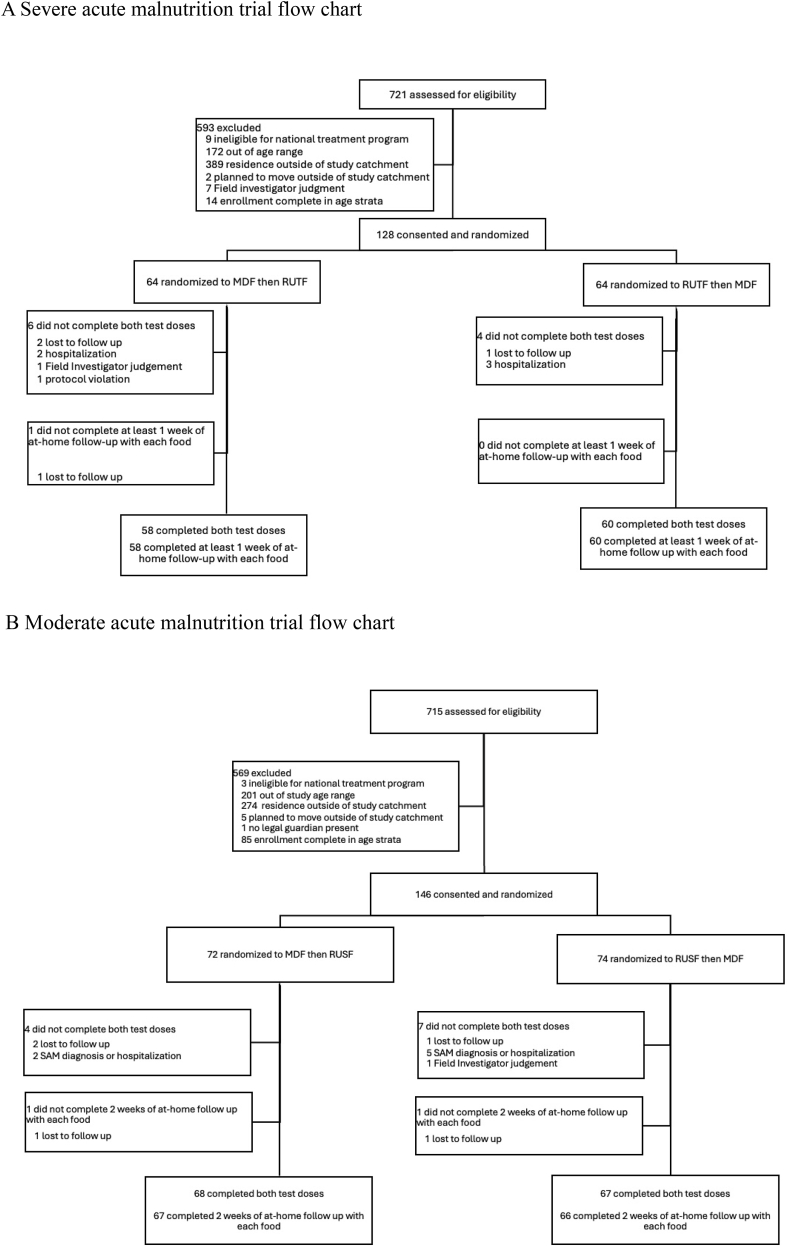
(A) Severe acute malnutrition trial flow chart. (B) Moderate acute malnutrition trial flow chart. MDF, microbiome-directed food; RUSF, ready-to-use supplementary food; RUTF, ready-to-use therapeutic food.

Between October 2022 and February 2023, 128 children with SAM and 146 children with MAM were enrolled (Figure 1A and B). In total, 118 children with SAM completed both observed test doses and ≥1 wk of at-home consumption with each food. For children with MAM, 135 completed both observed test doses and 133 completed 2 wk of at-home consumption with each food. Participant characteristics were generally well balanced between treatment sequences in both trials (Table 1). Nearly one-third (31.2%) of children with SAM and over one-quarter (26.7%) of children with MAM had been previously treated for acute malnutrition.

**TABLE 1:** Baseline child, maternal, and household characteristics stratified by severity and intervention sequence.

	Severe acute malnutrition		Moderate acute malnutrition	
	MDF then RUTF	RUTF then MDF	MDF then RUSF	RUSF then MDF
Children randomized, *n*	64	64	72	74
Sociodemographic characteristics				
Child’s age (mo), mean (SD)	14.1 (5.3)	13.5 (5.0)	12.8 (4.7)	12.4 (1.6)
Children aged 6–11 mo, *n* (%)	26 (40.6)	28 (43.8)	35 (48.6)	37 (50.0)
Children aged 12 to <24 mo, *n* (%)	38 (59.4)	36 (56.3)	37 (51.4)	37 (50.0)
Female sex, *n* (%)	31 (48.4)	31 (48.4)	39 (54.2)	34 (45.9)
Maternal level of education ≥6 y, *n* (%)	9 (14.1)	5 (7.8)	8 (11.1)	9 (12.2)
Household members, *n*, mean (SD)	8.9 (4.1)	9.5 (5.0)	10.2 (5.8)	10.2 (5.8)
HFIAS score, mean (SD)	8.2 (7.2)	7.9 (6.7)	6.7 (5.1)	6.1 (5.9)
Child anthropometric data				
Weight (kg), mean (SD)	6.2 (0.75)	6.1 (0.93)	6.9 (0.78)	6.9 (0.81)
Height (cm), mean (SD)	69.4 (4.7)	69.1 (5.1)	70.6 (4.4)	70.7 (4.5)
Weight-for-length *Z*-score, mean (SD)	−3.3 (0.74)	−3.3 (0.68)	−2.4 (0.41)	−2.4 (0.38)
Height-for-age *Z*-score, mean (SD)	−2.8 (1.6)	−2.7 (1.5)	−1.8 (1.3)	−1.6 (1.3)
Weight-for-age *Z*-score, mean (SD)	−3.8 (0.82)	−3.7 (0.89)	−2.7 (0.66)	−2.6 (0.7)
MUAC-for-age *Z*-score, mean (SD)	−3.1 (0.64)	−3.1 (0.68)	−2.0 (0.65)	−2.1 (0.64)
Mid-upper-arm circumference, mean (SD)	113.3 (6.0)	113.1 (6.5)	123.5 (6.1)	122.8 (6.3)
Breastfeeding status				
Currently breastfeeding, *n* (%)	44 (68.7)	50 (78.1)	60 (83.3)	64 (86.5)
Exclusively breastfeeding, *n* (%)	2 (0.03)	0 (0.0)	0 (0.0)	1 (1.4)

Abbreviations: HFIAS, Household Food Insecurity Access Scale; MDF, microbiome-directed food; RUSF, ready-to-use supplementary food; RUTF, ready-to-use therapeutic food.

### Acceptability among children with SAM

Overall, at-home consumption was high (≥94% of sachets consumed) for both study foods (Table 2). MDF was noninferior to RUTF in terms of at-home acceptability, with 93.2% of children with SAM returning ≥75% of MDF sachets empty compared with 100% returning ≥75% of RUTF sachets empty (RD: −7.0, 95% CI: lower bound −11.6%).

**TABLE 2:** Acceptability of MDF compared with standard nutritional care during 2 14-d at-home consumption periods, stratified by diagnosis severity.

	Severe acute malnutrition			Moderate acute malnutrition		
	MDF (*n* = 118)	RUTF (*n* = 118)		MDF (*n* = 133)	RUSF (*n* = 133)	
			Risk difference^1^ (95% CI lower bound)			Risk difference^1^ (95% CI lower bound)
Consumed >75% of provided sachets, *n* (%)	110 (93.2)	118 (100)	−7.0% (−11.6%)	128 (96.2)	131 (98.5)	−2.3% (−6.1%)

			Relative risk^2^ (95% CI)			Relative risk^2^ (95% CI)

Caregiver-reported child liking ≥ 4 on a 5-point scale, *n* (%)	112 (94.9)	118 (100)	0.95 (0.91, 0.99)	131 (98.5)	133 (100)	0.99 (0.96, 1.17)
Caregiver willingness to use food in future ≥ 4 on a 5-point scale, *n* (%)	116 (98.3)	117 (99.2)	0.99 (0.96, 1.02)	131 (98.5)	133 (100)	0.99 (0.96, 1.17)
Sharing of food with others, *n* (%)	2 (1.8)	0 (0.0)	Not estimable	1 (0.8)	3 (2.2)	0.33^2^ (0.04, 1.40)

			Mean difference (95% CI)			Mean difference (95% CI)

Percentage of dosage consumed over 2 wk, % (SD)	93.7 (11.2)	98.6 (3.5)	−4.9 (−6.9, −2.9)	95.6 (11.0)	98.8 (6.0)	−3.3 (−5.4, −1.2)
Times per day food consumed, mean (SD)	4.11 (1.38)	3.86 (1.11)	0.26 (0.01, 0.50)	2.47 (0.80)	2.40 (0.90)	0.07 (−0.09, 0.24)

Abbreviations: MDF, microbiome-directed food; RUSF, ready-to-use supplementary food; RUTF, ready-to-use therapeutic food.

1 Risk difference and one-sided confidence intervals (CIs) estimated using generalized estimating equations (GEE) with identity link and Poisson variance function. Relative risk and 95% CIs estimated using GEE with a log link and binomial variance function. All models included covariates for period and sequence effects and a compound symmetry covariance structure to account for the crossover design and covariates for sex and age group to account for stratified randomization.

2 Reduced model without adjustment for sex, age, and intervention sequence.

Secondary acceptability outcomes for the 14-d at-home consumption period for MDF and RUTF are presented in Table 2. Child’s liking of the products as perceived by the caregiver after at-home consumption was similarly high for the MDF (94.9%) and RUTF (100.0%) products, and there was no difference in caregiver willingness to use the food in future comparing MDF to RUTF (RR: 0.99; 0.96, 1.02). When caregivers were asked which food they preferred to use at the end of the 28-d follow-up period, 74.3% (81/109) preferred MDF compared with RUTF (28/109). In response to an open-ended question to explain this preference, caregivers reported a perception of more rapid weight gain and health improvement with MDF compared with RUTF. Mothers (25.7%) who preferred RUTF compared with MDF mentioned that RUTF was softer in texture and easier to consume.

Table 3 presents the acceptability of the observed test doses. We found no difference between MDF and RUTF in terms of observed test dose acceptability, with 59.3% of children with SAM consuming ≥75% of the MDF test dose compared with 56.8% consuming ≥75% of the RUTF test dose (RR: 1.06; 95% CI: 0.85, 1.33; Table 3). There was no difference in perceived child liking following the test dose, reason for completing the test dose, or time needed to complete the test dose between foods.

**TABLE 3:** Acceptability of MDF compared with standard nutritional care during a 30-min observed test dose, stratified by diagnosis severity.

	Severe acute malnutrition			Moderate acute malnutrition		
	MDF (*n* = 118)	RUTF (*n* = 118)		MDF (*n* = 135)	RUSF (*n* = 135)	
			Relative risk or mean difference^1^ (95% CI)			Relative risk or mean difference^1^ (95% CI)
Test dose acceptability,^2^*n* (%)	70 (59.3)	67 (56.8)	1.06 (0.85, 1.33)	62 (45.9)	79 (58.5)	0.80 (0.65, 0.98)
Liking ≥ 4 on Likert scale, *n* (%)	104 (88.1)	100 (84.7)	1.04 (0.94, 1.15)	116 (85.9)	122 (90.4)	0.95 (0.87, 1.05)
Reason for ending test dose						
Dose finished within 30 min, *n* (%)	49 (41.5)	48 (40.7)	1.07 (0.79, 1.45)	45 (33.3)	65 (48.1)	0.71 (0.56, 0.91)
Length of test dose (min), mean (SD)	12.2 (6.3)	12.8 (6.5)	−0.31 (−2.64, 2.01)	12.8 (6.7)	12.2 (7.2)	0.48 (−1.84, 2.80)
Time (30 min) elapsed, *n* (%)	32 (27.1)	35 (29.7)	0.91 (0.62, 1.33)	28 (20.7)	20 (14.8)	1.39 (0.84, 2.28)
Food refused 3 times, *n* (%)	37 (31.4)	35 (29.7)	1.34 (0.80, 2.24)	62 (45.9)	50 (37.0)	1.29 (0.99, 1.68)
Amount consumed (g), mean (SD)	14.2 (6.4)	14.2 (6.3)	−0.05 (−1.37, 1.27)	12.6 (6.8)	14.2 (6.7)	−1.55 (−2.84, −0.27)

Abbreviations: MDF, microbiome-directed food; RUSF, ready-to-use supplementary food; RUTF, ready-to-use therapeutic food.

1 Relative risk and 95% confidence intervals (CIs) estimated using generalized estimating equations (GEE) with a log link and binomial variance function. Mean difference and 95% CIs estimated using GEE with an identity link and Gaussian variance function. All models included covariates for period and sequence effects and a compound symmetry covariance structure to account for the crossover design and covariates for sex and age group to account for stratified randomization.

2 Test dose acceptability defined as consumption of 75% or more of the 20 g observed test dose.

### Acceptability among children with MAM

MDF was noninferior to RUSF in terms of at-home acceptability, with 96.2% of children with MAM returning ≥75% of MDF sachets empty compared with 98.5% returning ≥75% of RUSF sachets empty (RD: −2.3%; 95% CI: lower bound −6.1%; Table 2). There was no difference in child’s liking, caregiver willingness to use food in the future, or sharing comparing MDF with RUSF during at-home consumption.

Table 2 presents the results for secondary acceptability outcomes during the 14-d at-home consumption period. When asked their food preference after 4 wk of at-home consumption, 63.9% (85/133) caregivers preferred MDF compared with standard RUSF, describing fewer symptoms such as diarrhea and more rapid growth. Caregivers of children with MAM who preferred RUSF (36.1%; 48/133) stated that it was softer and easier to consume from the sachet.

The results of the observed test dose acceptability are presented in Table 3. MDF was less acceptable than RUSF during the observed test dose, with 45.9% of children with MAM consuming ≥75% of the MDF test dose compared with 58.5% consuming ≥75% of the RUSF test dose (RR: 0.80; 95% CI: 0.65, 0.98). Children were also less likely to complete the MDF test dose as compared with the RUSF test dose (RR: 0.71; 95% CI: 0.56, 0.91), but there was no difference in terms of perceived child liking during the observed test dose.

## Discussion

We conducted 2 randomized crossover trials to test the acceptability of a novel MDF compared with standard nutritional care in children with SAM and MAM in rural Niger. Acceptability was assessed during 2 14-d at-home consumption periods, as well as observed 30-min test doses. MDF was noninferior in terms of at-home acceptability when compared with standard nutritional care in both children with SAM and with MAM. Caregivers perceived MDF to be well-liked by children and reported no difference in their willingness to use either food in the future.

Standard RUTF is highly effective [27,28] and acceptable (83%–92% acceptability) across settings in Africa and Southeast Asia [29,30]. With the development of an increasing number of novel formulations of ready-to-use foods, UNICEF convened a technical expert meeting in 2019 to develop guidance for the scientific evaluation and assessment of alternative RUTF, defined as formulations that propose a change >10% in class of a bulk ingredient or that add a new ingredient class [31]. To enable new formulations to be ready for use in nutrition programs, criteria for assessment and trial designs for new formulations were proposed. The “model acceptability trial design” included a crossover study design that took place in a community setting over ≥4 wk in a population of children aged 6–24 mo with MAM or SAM with a sample size of 100–200 participants. This study followed this model design and provided the first evidence for this novel MDF to be acceptable among children aged 6 to <24 mo with SAM and MAM in a community setting in Niger. Other alternative formulations of RUTF using different bulk ingredients have similarly been evaluated using this study design, including a dairy-free RUTF in Zambia [32], a RUTF including groundnuts, soybean, and oats in Ethiopia [29], and a RUTF including soybean, maize, and coconut oil in Ghana [29], and been shown to be acceptable compared with standard nutritional care.

This study included an additional assessment method, an observed test dose, to further evaluate acceptability of a novel food at the time of introduction within a programmatic setting. Although acceptability was similar and high for all study foods during at-home consumption, MDF acceptability was somewhat lower than standard nutritional care during the observed test dose among children with MAM. Several studies have noted low acceptability during an observed test dose or initial phase that improved with continued home use over time, similar to our findings of greater acceptability during the longer at-home period compared with the initial test dose [30,[33], [34], [35]]. In a study in Bangladesh comparing 2 flavored lipid-based nutritional supplements in children, their acceptability differed significantly during an initial observed test dose, but there was no difference in acceptability during the 14-d at-home consumption period [34]. Children’s taste and acceptability of new foods, which can be initially low because of food neophobia (a reluctance to eat new or unfamiliar foods), can adapt as ongoing exposure leads to an increase in consumption. Provision of food for 8–10 d can increase acceptability in infants aged ≤24 mo [36], supporting the potential for greater acceptability of a novel MDF with continued exposure as expected in programmatic settings.

Our study had several strengths. First, the context allowed for examination of the acceptability of MDF in both children with MAM and children with SAM. MDF was developed in the context of MAM [13,14], and this study provides new evidence for its acceptability in children with both SAM or MAM, the ultimate target populations of such novel foods. Second, our study design included 2 complementary assessment methods, an observed test dose and at-home consumption. Acceptability was generally consistent across methods, allowing for greater certainty of findings. Third, our study included a complementary open-ended investigation to elicit caregiver preferences between study foods, allowing for feedback received on texture and ease of use that was shared with the manufacturer to improve future formulations. We also recognize several limitations of our study. First, at-home consumption was not directly observed. Sachet count is an imperfect proxy for acceptability but consistent with current guidelines for evaluation of novel RUTF formulations [19,37]. Second, the recommended crossover study design with 2 14 d at-home consumption periods is shorter than the maximum treatment duration of 12 wk, and child acceptability can change over time. Long-term acceptability should be monitored in further studies to ensure sustained acceptability and adherence over longer periods. Finally, we were unable to blind participants or study staff because of logistical constraints related to the package design (potentially resulting in participant expectation bias if caregivers were more likely to report positive results knowing the intervention received) nor allow for a washout period because of ethical considerations (potentially resulting in a carryover bias if the effects of the first food lingered into the subsequent consumption period).

Standard RUTF containing peanuts and milk powder has been shown to produce only relatively modest and transient repair of the perturbed (immature) microbiomes of children recovering from SAM in Bangladesh [12]. In 2 proof-of-concept clinical studies, the MDF used in this acceptability study significantly improved ponderal and linear growth in 12–18-mo-old Bangladeshi children with MAM, as well as in similarly aged children who were recovering after hospital-based treatment for SAM [14,15,38]. In addition to vegetable oil, sugar, and micronutrients, the MDF formulation includes chickpea flour, soybean flour, peanut paste, and green banana. Green banana and legumes (including chickpeas, soybeans, and peanuts) are high in resistant starch, a prebiotic demonstrated in prior studies to have several positive effects including enhanced mineral absorption and fat accumulation inhibition [39,40]. Moreover, a preclinical study demonstrated that resistant starch, added to the microbiome of preweaning and early weaning infants in Malawi in vitro, promoted acetate production, which may reduce gut inflammation and provide an additional energy source [41]. Owing to the representation of specific glycans present in its plant-based ingredients [42,43], the MDF used here has been shown to induce superior repair of the disrupted microbiota of acutely malnourished Bangladeshi children and produce not only improvements in ponderal and linear growth [14,15,38], but also increases in the levels of plasma proteins linked to musculoskeletal- and neurodevelopment [14,42,43]. These data suggest that this MDF may be a promising new formulation to improve long-term growth and development by targeting microbiome repair.

In conclusion, we found MDF to be noninferior to standard nutritional care among children with SAM and MAM in terms of at-home acceptability. This study sets the stage for a large-scale randomized controlled effectiveness trial to assess the generalizability of the benefits of MDF in children aged 6–24 mo compared with standard nutritional care in settings outside Bangladesh (clinicaltrials.gov identifier: NCT06382857). Our trial in Niger will monitor nutritional recovery during treatment and sustained recovery following treatment, and through longitudinal fecal/blood collection, measure the effects of the interventions on microbiome repair and their effects on levels of plasma biomarkers of growth and development.

## Author contributions

The authors’ responsibilities were as follows – SI, IM, KNS, OG, TA, MJB, JIG, RFG: conceptualized the study; SI: designed the study and data collection instruments; INM, MAAT, SG: conducted the research; SMR, CRS: analyzed data; SMR, SI: wrote the initial draft; SI: had primary responsibility for final content; and all authors: reviewed and revised the manuscript for important intellectual content and approved the final manuscript.

## Data availability

Data described in the manuscript, code book, and analytic code will be made available on request. Researchers interested in accessing the data should submit a formal data access request to the corresponding author, outlining their research objectives and ensuring compliance with relevant ethical guidelines.

## Funding

These trials were funded by the Bill & Melinda Gates Foundation (INV-023315).

## Conflict of interest

SI reports financial support was provided by Bill and Melinda Gates Foundation. The other authors report no conflicts of interest.

## References

[1] Wright C.M., Macpherson J., Bland R., Ashorn P., Zaman S., Ho F.K. (2021). Wasting and stunting in infants and young children as risk factors for subsequent stunting or mortality: longitudinal analysis of data from Malawi, South Africa, and Pakistan. J. Nutr..

[2] UNICEF, WHO, World Bank Group (2023).

[3] (2023). WHO guideline on the prevention and management of wasting and nutritional oedema (acute malnutrition) in infants and children under 5 years.

[4] World Vision (2017).

[5] Somasse Y.E., Bahwere P., Laokri S., Elmoussaoui N., Donnen P. (2013). Sustainability and scaling-up analysis of community-based management of acute malnutrition: lessons learned from Burkina Faso, Food Nutr. Bull.

[6] Aguayo V.M., Badgaiyan N., Qadir S.S., Bugti A.N., Alam M.M., Nishtar N. (2018). Community management of acute malnutrition (CMAM) programme in Pakistan effectively treats children with uncomplicated severe wasting, Matern. Child Nutr..

[7] Sanchez-Martinez L.J., Charle-Cuellar P., Gado A.A., Dougnon A.O., Sanoussi A., Ousmane N. (2023). Impact of a simplified treatment protocol for moderate acute malnutrition with a decentralized treatment approach in emergency settings of Niger. Front. Nutr..

[8] Stobaugh H.C., Bollinger L.B., Adams S.E., Crocker A.H., Grise J.B., Kennedy J.A. (2017). Effect of a package of health and nutrition services on sustained recovery in children after moderate acute malnutrition and factors related to sustaining recovery: a cluster-randomized trial. Am. J. Clin. Nutr..

[9] Stobaugh H.C., Mayberry A., McGrath M., Bahwere P., Zagre N.M., Manary M.J. (2019). Relapse after severe acute malnutrition: a systematic literature review and secondary data analysis. Matern. Child Nutr..

[10] Blanton L.V., Charbonneau M.R., Salih T., Barratt M.J., Venkatesh S., Ilkaveya O. (2016). Gut bacteria that prevent growth impairments transmitted by microbiota from malnourished children. Science.

[11] Raman A.S., Gehrig J.L., Venkatesh S., Chang H.W., Hibberd M.C., Subramanian S. (2019). A sparse covarying unit that describes healthy and impaired human gut microbiota development. Science.

[12] Subramanian S., Huq S., Yatsunenko T., Haque R., Mahfuz M., Alam M.A. (2014). Persistent gut microbiota immaturity in malnourished Bangladeshi children. Nature.

[13] Gehrig J.L., Venkatesh S., Chang H.W., Hibberd M.C., Kung V.L., Cheng J. (2019). Effects of microbiota-directed foods in gnotobiotic animals and undernourished children. Science.

[14] Chen R.Y., Mostafa I., Hibberd M.C., Das S., Mahfuz M., Naila N.N. (2021). A microbiota-directed food intervention for undernourished children. N. Engl. J. Med..

[15] Mostafa I., Hibberd M.C., Hartman S.J., Hafizur Rahman M.H., Mahfuz M., Hasan S.M.T. (2024). A microbiota-directed complementary food intervention in 12-18-month-old Bangladeshi children improves linear growth. EBioMedicine.

[16] UNDP (United Nations Development Programme) (2024). Poverty amid conflict.

[17] Institut National de la Statistique Niger (2022).

[18] Ministère de la Santé Publique Niger (2016).

[19] UNICEF (2019).

[20] Likert R. (1932). A technique for the measurement of attitudes. Arch. Psychol..

[21] Pedroza C., Truong V.T. (2016). Performance of models for estimating absolute risk difference in multicenter trials with binary outcome. BMC Med. Res. Methodol..

[22] Zou G. (2004). A modified Poisson regression approach to prospective studies with binary data. Am. J. Epidemiol..

[23] Li T., Yu T., Hawkins B.S., Dickersin K. (2015). Design, analysis, and reporting of crossover trials for inclusion in a meta-analysis. PLOS ONE.

[24] Lim C.Y., In J. (2021). Considerations for crossover design in clinical study. Korean J. Anesthesiol..

[25] Simon L.J., Chinchilli V.M. (2007). A matched crossover design for clinical trials. Contemp. Clin. Trials..

[26] Kahan B.C., Morris T.P. (2012). Reporting and analysis of trials using stratified randomisation in leading medical journals: review and reanalysis. BMJ.

[27] Maleta K., Amadi B. (2014). Community-based management of acute malnutrition (CMAM) in sub-Saharan Africa: case studies from Ghana, Malawi, and Zambia, Food Nutr. Bull.

[28] WHO, WFP, UNSCN, UNICEF (2007). Joint statement on community-based management of severe acute malnutrition. http://www.who.int/maternal_child_adolescent/documents/a91065/en/.

[29] Weber J.M., Ryan K.N., Tandon R., Mathur M., Girma T., Steiner-Asiedu M. (2017). Acceptability of locally produced ready-to-use therapeutic foods in Ethiopia, Ghana, Pakistan and India, Matern. Child Nutr..

[30] Nga T.T., Nguyen M., Mathisen R., Hoa do T.B., Minh N.H., Berger J. (2013). Acceptability and impact on anthropometry of a locally developed ready-to-use therapeutic food in pre-school children in Vietnam. Nutr. J..

[31] Fleet A., Kshirsagar R., Bach M., Forteza M. (2019).

[32] Owino V.O., Irena A.H., Dibari F., Collins S. (2014). Development and acceptability of a novel milk-free soybean-maize-sorghum ready-to-use therapeutic food (SMS-RUTF) based on industrial extrusion cooking process, Matern. Child Nutr..

[33] Aiello I., Kounnavong S., Vinathan H., Philavong K., Luangphaxay C., Soukhavong S. (2023). Short-term acceptability of ready-to-use therapeutic foods in two provinces of Lao People's Democratic Republic. Nutrients.

[34] Mridha M.K., Matias S.L., Chaparro C.M., Paul R.R., Hussain S., Vosti S.A. (2016). Lipid-based nutrient supplements for pregnant women reduce newborn stunting in a cluster-randomized controlled effectiveness trial in Bangladesh. Am. J. Clin. Nutr..

[35] Sigh S., Roos N., Sok D., Borg B., Chamnan C., Laillou A. (2018). Development and acceptability of locally made fish-based, ready-to-use products for the prevention and treatment of malnutrition in Cambodia. Food Nutr. Bull..

[36] Spill M.K., Johns K., Callahan E.H., Shapiro M.J., Wong Y.P., Benjamin-Neelon S.E. (2019). Repeated exposure to food and food acceptability in infants and toddlers: a systematic review. Am. J. Clin. Nutr..

[37] Tondeur M.C., Salse U.N., Wilkinson C., Spiegel P., Seal A.J. (2016). Rapid acceptability and adherence testing of a lipid-based nutrient supplement and a micronutrient powder among refugee children and pregnant and lactating women in Algeria. Public Health Nutr.

[38] Hartman S.J., Hibberd M.C., Mostafa I., Naila N.N., Islam M.M., Zaman M.U. (2024). A microbiome-directed therapeutic food for children recovering from severe acute malnutrition. Sci. Transl. Med..

[39] DeMartino P., Cockburn D.W. (2020). Resistant starch: impact on the gut microbiome and health. Curr. Opin. Biotechnol..

[40] Sajilata M.G., Singhal R.S., Kulkarni P.R. (2006). Resistant starch-A review. Compr. Rev. Food Sci. Food Saf..

[41] Wang Y., Mortimer E.K., Katundu K.G.H., Kalanga N., Leong L.E.X., Gopalsamy G.L. (2019). The capacity of the fecal microbiota from Malawian infants to ferment resistant starch. Front. Microbiol..

[42] Chang H.W., Lee E.M., Wang Y., Zhou C., Pruss K.M., Henrissat S. (2024). Prevotella copri and microbiota members mediate the beneficial effects of a therapeutic food for malnutrition. Nat. Microbiol..

[43] Hibberd M.C., Webber D.M., Rodionov D.A., Henrissat S., Chen R.Y., Zhou C. (2024). Bioactive glycans in a microbiome-directed food for children with malnutrition. Nature.

